# Benign cystic mesothelioma: A case report on the presentation of an unusual tumor

**DOI:** 10.1016/j.ijscr.2021.105918

**Published:** 2021-04-30

**Authors:** Yousif Salem, Abeer Farhan, Hasan S. Aljawder, Abdulmenem Abualsel

**Affiliations:** King Hamad University Hospital, Bahrain

**Keywords:** Benign cystic mesothelioma, Laparoscopic appendectomy, Inclusion cyst, Abdominal tumors, Case report

## Abstract

**Introduction:**

Benign cystic mesothelioma of the peritoneum is a rare, benign abdominal tumor. It can present with vague signs and symptoms and is often found on imaging or incidentally during surgery.

**Presentation of case:**

We report the case of a 30-year-old man presenting with acute abdominal pain that radiated to the right iliac fossa. No masses were found on superficial or deep palpation or on conducting a sonography. The patient underwent a diagnostic laparoscopy with an appendectomy, which revealed a perforated appendix and two cysts in the pelvis and iliac fossa.

**Discussion:**

A benign cystic mesothelioma is an inclusion cyst found in the peritoneal cavity and has no specific clinical presentation. It can be symptomatic or found incidentally during surgery. Benign cystic mesotheliomas have a high recurrence rate and may undergo malignant transformation.

**Conclusion:**

Complete surgical excision of benign cystic mesothelioma must always be the first step of the treatment plan for this condition. It is difficult to treat with no evidence-based treatment modality available; thus, treatment should only be undertaken in a specialized center.

## Highlights

• Benign cystic mesothelioma of the peritoneum is presents with vague symptoms.
• It can be symptomatic or found incidentally during surgery.
• To date, only 140 cases have been reported in the literature.
• Diagnosis is generally made via microscopic examination of the specimen.
• Mainstay of treatment for this tumor is complete surgical excision.

## 1 Introduction

A benign cystic mesothelioma of the peritoneum is considered a rare entity, as only 140 cases have been reported in the literature since 1979, when Smith and Mennenmeyer described the first case. Herein, we present a case of benign cystic mesothelioma that was incidentally found during laparoscopic appendectomy. Our aim is to understand the presentation and common features of the entity by correlating our case with those reported in the literature.

## 2 Presentation of case

A 30-year-old man with no significant medical or surgical history presented to our hospital with one day history of severe epigastric pain radiating to the right iliac fossa which was partially relieved with analgesia, associated with nausea and multiple episodes of non-bilious vomiting.

Upon clinical examination, the patient was stable. The abdomen was soft with tenderness over McBurney's point, and there were no masses on superficial or deep palpation.

Laboratory investigations showed a white cell count of 11,500 cells/L with 72% neutrophils and a C-reactive protein level of 10.5 mg/l. In combination with the patient's clinical examination, the findings were in line with the criteria for acute appendicitis.

**Fig. 1:**
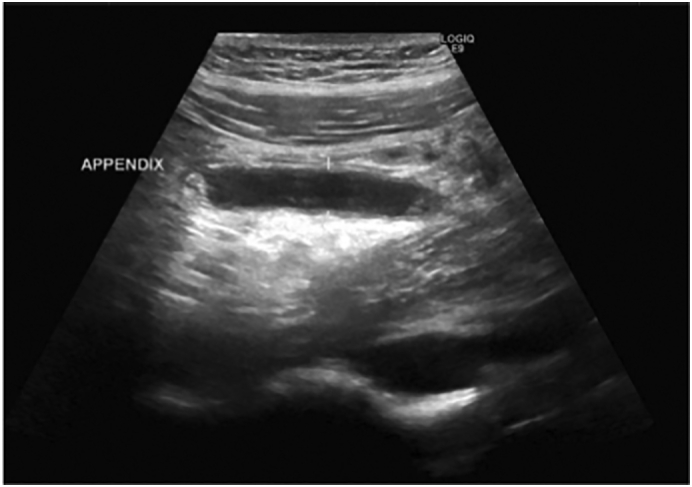
Ultrasonic image showing non-compressible structure at the RIF (Appendix).

Abdominal ultrasound revealed a mildly distended, non-compressible appendix measuring 10 mm in diameter, with an echogenic shadow at the base and the collection of a small amount of fluid in the right iliac fossa (Fig. 1). No intra-abdominal masses were detected on sonography.

**Fig. 2:**
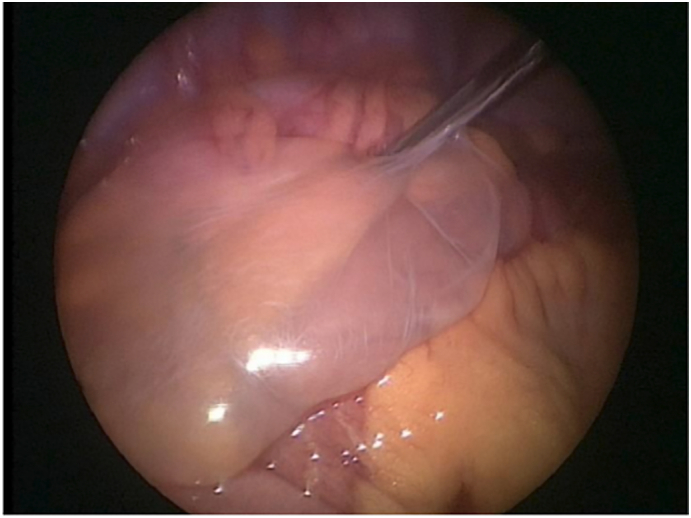
Intraoperative image of the cystic lesion.

The patient underwent diagnostic laparoscopy with the intention of doing laparoscopic appendectomy. Intraoperatively, the appendix was found to be inflamed, hence appendectomy ensued. Upon further exploration two cysts that contained gelatinous material found in the pelvis and left iliac fossa incidentally. The cysts and the appendix were excised simultaneously and sent for histopathology. Neither cyst was attached to the intra-abdominal organs or to the abdominal wall (Fig. 2).

Postoperatively, the patient received adequate analgesics and intravenous antibiotics. The hospital course was uneventful, and the patient was discharged in a stable condition.

**Fig. 3:**
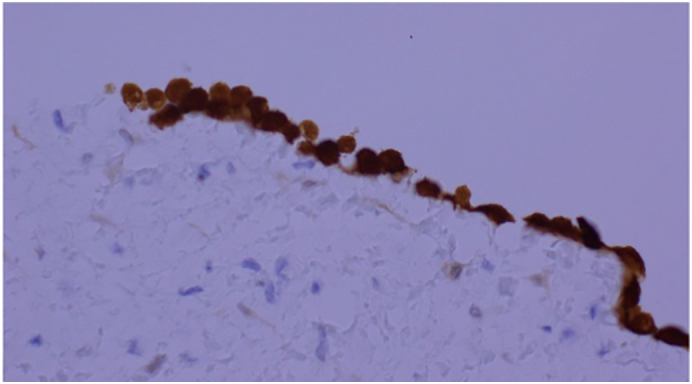
Histopathological image showing calretinin positivity of the lining cells.

Histopathology confirmed an inflamed, non-perforated appendix and a multicystic benign mesothelioma, which was positive for calretinin on immunohistochemistry (Fig. 3).

Regular postoperative follow-ups were performed in the clinic 2 weeks, 3 months, and 1 year after surgery. The surgical wounds healed naturally with no complications. The patient resumed normal activities and returned to work 1 week after surgery.

## 3 Discussion

Benign cystic mesotheliomas are inclusion cysts found in the peritoneal cavity that cause vague signs and symptoms, including abdominal pain, a palpable abdominal mass, and abdominal distention. Occasionally, they are found incidentally during laparotomy [1,2]. They commonly arise from the peritoneal mesothelial lining and contain fluid material. Benign cystic mesotheliomas sometimes contain gaseous and semisolid material, in which case, they are considered a variant of intra-abdominal lymphangiomas [2] and mimic ovarian malignancies. Electron microscopy is used along with immunohistochemistry to distinguish these different types from each other [3]. Although their exact etiology is obscured; they are thought to be due to.

Benign cystic mesotheliomas are more common in women than in men, and among women, are more common in individuals of reproductive age. Clinical presentation does not differ between the sexes [3,4].

Benign cystic mesotheliomas are rare. To date, only 140 cases have been reported in the literature after being first described in 1979 by Smith and Mennenmeyer [5].

There is no specific clinical presentation of this condition. It can be symptomatic or, as mentioned previously, found incidentally during surgery, similar to what happened in our case. Symptoms can range from vague to severe abdominal pain, the presence of a palpable abdominal mass, or pelvic discomfort [3,4].

A diagnosis of benign cystic mesothelioma is made mainly via microscopic examination of the specimen, which shows multiple cystic structures lined with the mesothelium and a fibromuscular stroma. Immunohistochemical staining with calretinin and cytokeratin 5/6 is also used for diagnosis, as these stains are specific to mesothelial cells and act as positive markers in the diagnosis of mesothelioma [5,6].

Benign cystic mesotheliomas are localized tumors without the potential for metastasis. Despite their benign nature, they have the potential to recur [1] and carry a risk of malignant transformation; hence, many consider these cysts as borderline tumors [4]. Mortality has been reported in a 14-year-old patient who underwent a subtotal resection of the mass but died 12 years after recurrence and refusal of surgery [7].

## 4 Conclusion

In this study, we examined a case of benign cystic mesothelioma in a stable patient with no significant medical history.

A benign cystic mesothelioma is a rare benign tumor with a high recurrence rate and potential malignant transformation, and close and regular follow-up for patients of this condition is highly recommended. As there is no evidence-based treatment modality, there is a need for more cases of benign cystic mesothelioma to be reported in the literature.

Based on the previously reported cases, this tumor requires optimal care in a specialized center. The mainstay of the treatment of this condition is complete surgical excision of the mass.

## Patient perspective

The patient participated in the treatment decision and he was satisfied with the results of the treatment. His perspective on this treatment was to be treated without complications.

## Provenance and peer review

Not commissioned, externally peer-reviewed.

## Sources of funding

This research did not receive any specific grant from funding agencies in the public, commercial, or not-for-profit sectors.

## Ethical approval

None required.

## Consent

Written informed consent was obtained from the patient for publication of this case report and accompanying images. A copy of the written consent is available for review by the Editor-in-Chief of this journal on request.

## Research registration

None declared.

## Guarantor

Dr. Yousif Salem

## CRediT authorship contribution statement

Yousif Salem: Was involved in drafting the article and revising it critically for important intellectual content.

Abeer Farhan: Was involved in conception and design, acquisition of data and revising it critically for important intellectual content.

Hasan S. Aljawder: provided substantial contribution to the conception and design of the written report and acquisition of photographs.

Abdulmenem Abualsel: Was involved in final approval of the version published and agreement that all questions regarding the accuracy or integrity of the article are investigated and resolved.

## Declaration of competing interest

None.
